# Association between gallstones and arthritis in U.S. adults: A cross-sectional NHANES study

**DOI:** 10.1016/j.pmedr.2025.103335

**Published:** 2025-12-02

**Authors:** Junyu Huang, Zan Liu

**Affiliations:** a Department of Gastroenterology, Hui Ya Hospital of The First Affiliated Hospital, Sun Yat-sen University, Huizhou, China; b Department of Health Management Center, Hui Ya Hospital of The First Affiliated Hospital, Sun Yat-sen University, Huizhou, China

**Keywords:** Gallstones, Arthritis, NHANES, Cross-sectional study, Logistic regression

## Abstract

**Objective:**

This study aimed to investigate the association between gallstones and arthritis, including osteoarthritis and rheumatoid arthritis, in U.S. adults.

**Methods:**

A cross-sectional analysis was conducted using National Health and Nutrition Examination Survey data from 2017 to 2020 and 2021–2023, including 10,108 participants. Weighted multivariable logistic regression models were employed to analyze the association, adjusting for demographic, lifestyle, and metabolic confounders. Subgroup and interaction analyses were performed to assess effect modification of categorical covariates.

**Results:**

Gallstones were significantly associated with arthritis (OR: 1.49, 95 % CI: 1.15, 1.92) and osteoarthritis (OR: 1.55, 95 % CI: 1.15, 2.08) after full adjustment, but not with rheumatoid arthritis. Subgroup and interaction analyses revealed a significant effect modification by sex (P for interaction = 0.02), with females showing a significantly stronger association between gallstones and osteoarthritis (OR: 1.81, 95 % CI: 1.33, 2.47).

**Conclusions:**

Gallstones were significantly associated with osteoarthritis, particularly among females. Further studies are needed to clarify the causal association.

## Highlights

• Gallstones are independently associated with osteoarthritis in U.S. adults.
• The gallstones–osteoarthritis link is stronger in females, indicating sex-specific modification.
• Gallstones show no significant association with rheumatoid arthritis after full adjustment.

## 1 Background

Arthritis is a group of chronic diseases characterized primarily by joint inflammation, pain, and functional impairment, encompassing various subtypes, the most common of which are osteoarthritis and rheumatoid arthritis(Lewis et al., 2019). The etiology of arthritis is complex, involving multifactorial interactions such as genetic, metabolic, immune, mechanical load, and inflammatory factors(Husaini et al., 2025). This chronic condition is associated with significant morbidity, reduced quality of life, and considerable economic costs due to long-term treatment requirements and productivity losses. The global prevalence continues to rise with aging populations, making arthritis a pressing public health challenge that demands effective prevention and management strategies(Guan et al., 2023). Current studies have revealed the associative mechanisms between arthritis and other systemic diseases, which may provide new insights for disease prevention and treatment(Basak et al., 2023; S. Gao et al., 2025a; Gong and Baek, 2025; Que et al., 2025).

Gallstones are a highly prevalent digestive disorder worldwide, classified into asymptomatic gallstones, acute calculous cholecystitis and chronic calculous cholecystitis, involving mechanisms such as cholesterol metabolic dysfunction, biliary stasis, inflammatory responses and so on(K. Wang et al., 2025b). Although some patients remain asymptomatic for life, they frequently demonstrate a persistent low-grade inflammatory status(Psaltis et al., 2024), which may contribute to immune-mediated diseases through shared pathophysiological pathways. Arthritis, a common chronic inflammatory disorder, is also closely associated with metabolic disturbances and autoimmune reactions. Previous studies have revealed a potential link between gallstones and arthritis, but these investigations were limited by small sample sizes, possibly resulting in diminished statistical power(García-Gómez et al., 2019; Pamuk et al., 2006). Furthermore, the potential modifying effects of key demographic and metabolic factors on this association remain unclear. The prevalence of both gallstones and arthritis is strongly influenced by sex, age, and obesity status (Lewis et al., 2019; K. Wang et al., 2025b). For instance, gallstones are more prevalent in females, partly due to hormonal influences on bile metabolism(Ismail et al., 2023). Given that these are shared risk factors, we hypothesized that sex, age, and body mass index (BMI) might significantly modify the association between gallstones and arthritis. Therefore, our study utilized the National Health and Nutrition Examination Survey (NHANES), a database with its large sample size and representative sample, to further investigate the association between gallstones and arthritis. We also aimed to explore whether this association is consistent across strata defined by these and other important covariates.

## 2 Methods

### 2.1 Study design and population

This cross-sectional analysis utilized data from NHANES 2017–2020 and 2021–2023 cycles. NHANES is a well-designed population-based study aimed at assessing the nutritional status and general health of the non-institutionalized US population. Conducted by the National Center for Health Statistics across multiple sites, it employs a stratified, multistage probability sampling methodology to ensure nationally representative estimates. NHANES performs biannual cross-sectional surveys, with each cycle encompassing approximately 50,000 U.S. residents, and the protocol was approved by the Ethics Review Board of the National Center for Health Statistics. Detailed NHANES datasets and documentation are available on the official website (https://www.cdc.gov/nchs/nhanes/about_nhanes.htm). All participants provided written informed consent. Thus, no additional ethical approval was required for this study.

**Fig. 1:**
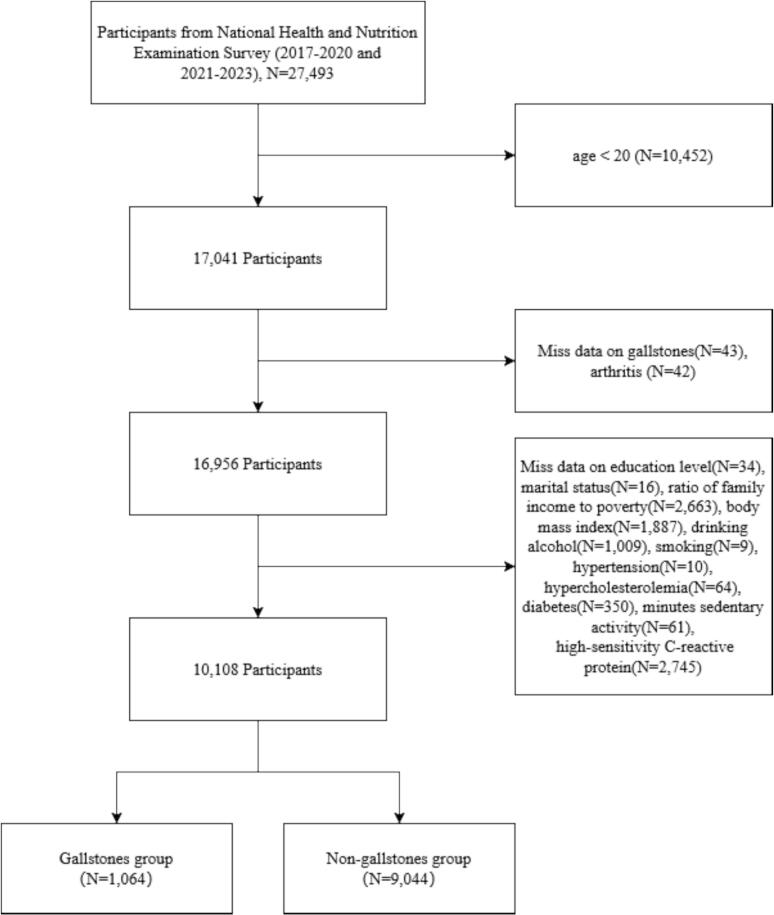
Flow chart of participant selection for the cross-sectional study on gallstones and arthritis using data from the National Health and Nutrition Examination Survey, 2017–2020 and 2021–2023.

We analyzed data from 27,493 participants. The following exclusion criteria were applied: age < 20 years; missing gallstone questionnaire data; missing arthritis questionnaire data; missing covariate data. After exclusions, 10,108 participants were included in the final analysis, of whom 1064 (10.5 %) had gallstones and 9044 (89.5 %) did not. The data inclusion and exclusion criteria are illustrated in Fig. 1.

### 2.2 Measures

#### 2.2.1 Exposure variable

The exposure variable in this study was gallstones. Gallstones were assessed via questionnaire based on participants' responses to the item, ‘Has a doctor or other health professional ever told you that you had gallstones?’ Participants who affirmed this diagnosis were classified as having gallstones, while those who denied such diagnosis were classified as non-gallstones(Chen et al., 2024).

#### 2.2.2 Outcome variable

In this study, arthritis served as the outcome variable. Assessment was conducted via questionnaire using two items: (1) ‘Has a doctor or other health professional ever told you that you had arthritis?’ and (2) ‘Which type of arthritis was it?’ Participants who responded “yes” to the first question were classified as having arthritis, and were further categorized by arthritis type based on their response to the second question(He et al., 2025).

#### 2.2.3 Covariates

The covariates that may influence the association between gallstones and arthritis were selected in this study as follows: (1) Demographic characteristics included age, sex, race, education level, marital status, family poverty-income ratio (PIR), and body mass index (BMI); (2) Lifestyle factors included smoking status, alcohol consumption, and sedentary activity; (3) Health conditions included hypertension, diabetes, hypercholesterolemia, and high-sensitivity C-reactive protein levels. Race was categorized as Mexican American, other Hispanic, non-Hispanic White, non-Hispanic Black, and other. Educational level was dichotomized into under high school and High school or above. Marital status included married/living with partner, widowed/divorced/separated, and never married. PIR served as an economic status indicator, with PIR < 1 indicating poverty and PIR ≥ 1 indicating non-poverty(Montiel Ishino et al., 2022). Smoking status was defined as having smoked >100 cigarettes in lifetime(X. Gao et al., 2025b). Alcohol consumption was defined as ever had a drink of any kind of alcohol(X. Gao et al., 2025b). Sedentary behavior was measured as daily sedentary time, with <360 min classified as non-sedentary activity and ≥ 360 min as sedentary activity(Cao et al., 2022). Hypertension, diabetes, and hypercholesterolemia were ascertained through self-reported physician diagnoses per NHANES questionnaire protocols. High-sensitivity C-reactive protein levels were obtained via laboratory assays.

### 2.3 Statistical analysis

To account for the complex survey design and ensure nationally representative estimates, we applied appropriate sampling weights in all analyses. Participants were stratified by gallstones (yes/no). Continuous variables were expressed as mean ± standard deviation, while categorical variables were reported as percentages (%). Differences in baseline characteristics between groups were assessed using Student's *t*-tests for continuous variables and chi-square tests for categorical variables. The association between gallstones and arthritis, included its subtypes, was evaluated using multivariable logistic regression models, with results presented as odds ratio (OR) and 95 % confidence interval (95 % CI). Additionally, we conducted stratified subgroup analyses and tested for potential interaction effects. All analyses were conducted using R software (version 4.3.1). Statistical significance was recognized when *P* < 0.05.

## 3 Results

### 3.1 Participant characteristics

The characteristics of the participants were presented in Table 1. Statistically significant differences were observed between the two groups in terms of age, sex, BMI, race, marital status, smoking status, hypertension, diabetes, hypercholesterolemia, and high-sensitivity C-reactive protein levels. The gallstones group was significantly older, had a higher proportion of females, a higher BMI, and a higher percentage of smokers. Moreover, the prevalence of comorbidities such as hypertension, diabetes, and hypercholesterolemia was higher in this group. Notably, the prevalence of arthritis and its subtypes (osteoarthritis and rheumatoid arthritis) was significantly higher in the gallstones group than in the non-gallstones group (50.9 % vs 25.5 %, 30.4 % vs 13.1 %, and 7.1 % vs 4.0 %; all *P* < 0.01).

**Table 1:** Distribution of characteristics among U.S. adults with and without gallstones: data from the National Health and Nutrition Examination Survey, 2017–2020 and 2021–2023.

Characteristic	Overall	Gallstones	Non-gallstones	*P* value
n	**10,108**	**1064**	**9044**	
Age, year	48.4 ± 17.2	57.3 ± 15.3	47.5 ± 17.1	<0.01
Sex(%)				<0.01
Male	48.9	25.8	51.4	
Female	51.1	74.2	48.6	
BMI, kg/m^2^	29.7 ± 7.1	33.4 ± 8.5	29.3 ± 6.8	<0.01
Race(%)				0.02
Mexican American	6.9	6.3	7.0	
Other Hispanic	7.7	8.3	7.7	
Non-Hispanic White	65.3	69.5	64.7	
Non-Hispanic Black	9.9	6.1	10.3	
Other Race	10.2	9.8	10.3	
Education level(%)				0.50
Under high school	8.7	9.6	8.6	
High school or above	91.3	90.4	91.4	
Marital status(%)				<0.01
Married/Living with Partner	62.0	61.4	62.1	
Widowed/Divorced/Separated	18.5	26.7	17.6	
Never married	19.5	11.9	20.3	
PIR(%)				0.70
<1	12.0	12.6	11.9	
≥1	88.0	87.4	88.1	
Alcohol consumption(%)				0.70
Yes	92.6	92.2	92.6	
No	7.4	7.8	7.4	
Smoke status(%)				<0.01
Yes	39.9	48.1	39.1	
No	60.1	51.9	60.9	
Sedentary activity,h				0.20
<6	50.8	48.0	51.1	
≥6	49.2	52.0	48.9	
Hypertension(%)				<0.01
Yes	30.5	47.6	28.7	
No	69.5	52.4	71.3	
Diabetes(%)				<0.01
Yes	11.4	20.6	10.4	
No	88.6	79.4	89.6	
Hypercholesterolemia(%)				<0.01
Yes	35.0	51.4	33.2	
No	65.0	48.6	66.8	
Arthritis(%)				<0.01
Yes	27.9	50.9	25.5	
No	72.1	49.1	74.5	
Arthritis type(%)				<0.01
No	72.1	49.1	74.5	
Osteoarthritis	14.7	30.4	13.1	
Rheumatoid arthritis	4.3	7.1	4.0	
Others	8.9	13.4	8.4	
high-sensitivity C-reactive protein, mg/L	3.8 ± 7.2	5.3 ± 8.6	3.6 ± 7.0	<0.01

Values are presented as mean ± standard deviation or percentage (%). *P* values were derived from Student's t-tests for continuous variables and chi-square tests for categorical variables.

Abbreviations: BMI, body mass index; PIR, poverty-income ratio.

### 3.2 Association between gallstones and arthritis

We investigated the association between gallstones and arthritis, including its subtypes (osteoarthritis and rheumatoid arthritis) using weighted multivariable logistic regression models, with detailed results shown in Table 2. In the unadjusted Model 1, gallstones were significantly associated with arthritis (OR: 3.02, 95 % CI: 2.44, 3.75), osteoarthritis (OR: 3.51, 95 % CI: 2.66, 4.62), and rheumatoid arthritis (OR: 2.69, 95 % CI: 1.82, 3.96). After adjusting for age, sex, and race in Model 2, these associations remained significant for arthritis (OR: 1.90, 95 % CI: 1.48, 2.44), osteoarthritis (OR: 1.99, 95 % CI: 1.49, 2.67), and rheumatoid arthritis (OR: 1.77, 95 % CI: 1.11, 2.81). Further adjustment for education level, marital status, PIR, BMI, alcohol consumption, smoking status, hypertension, hypercholesterolemia, diabetes, and sedentary activity in Model 3 revealed that gallstones maintained significant associations with arthritis (OR: 1.49, 95 % CI: 1.15, 1.92) and osteoarthritis (OR: 1.55, 95 % CI: 1.15, 2.08), while the association with rheumatoid arthritis became non-significant (OR: 1.45, 95 % CI: 0.89, 2.36).

**Table 2:** Associations between gallstones and arthritis among U.S. adults from the National Health and Nutrition Examination Survey, 2017–2020 and 2021–2023.

Outcome	Model 1 OR (95 %CI)	Model 2 OR (95 %CI)	Model 3 OR (95 %CI)
Non-arthritis	Ref	Ref	Ref
Arthritis	3.02 (2.44, 3.75)	1.90 (1.48, 2.44)	1.49 (1.15, 1.92)
osteoarthritis	3.51 (2.66, 4.62)	1.99 (1.49, 2.67)	1.55 (1.15, 2.08)
rheumatoid arthritis	2.69 (1.82, 3.96)	1.77 (1.11, 2.81)	1.45 (0.89, 2.36)

Model 1 was not adjusted for any covariates.

Model 2 was adjusted for age, gender, race.

Model 3 was adjusted for age, gender, race, education, marital status, PIR, BMI, drinking status, smoking status, sedentary activity, hypertension, diabetes and hypercholesterolemia.

Abbreviations: OR, odds ratio; CI, confidence interval; Ref, reference; BMI, body mass index; PIR, poverty-income ratio.

### 3.3 Subgroup analysis

We conducted subgroup analyses stratified by age, sex, BMI, sedentary behavior, smoking status, alcohol consumption, diabetes, hypertension, and hyperlipidemia.

**Fig. 2:**
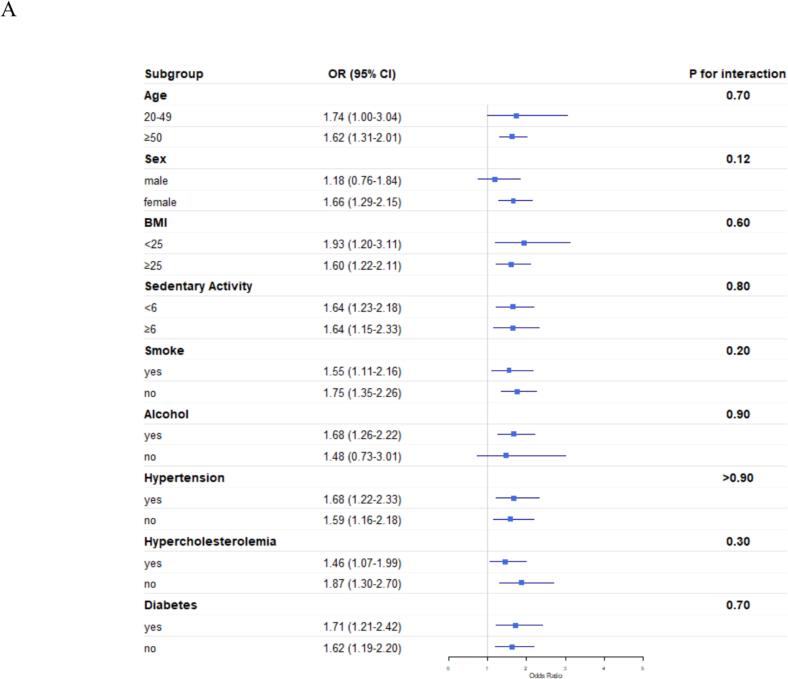

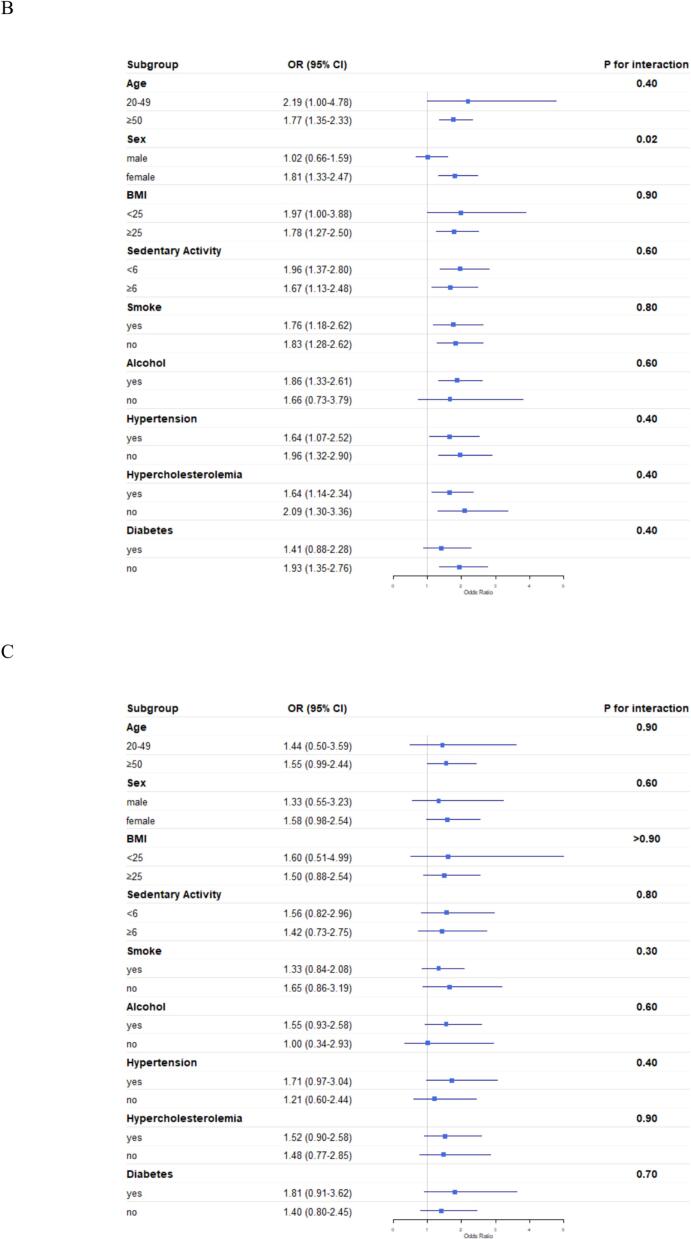
Subgroup analyses of the association between gallstones and (A) arthritis, (B) osteoarthritis, and (C) rheumatoid arthritis among U.S. adults from the National Health and Nutrition Examination Survey, 2017–2020 and 2021–2023.•Abbreviations: BMI, body mass index; OR, odds ratio; CI, confidence interval.

As shown in Fig. 2A, significant positive associations between gallstones and arthritis were consistently observed across all subgroups stratified by BMI, sedentary behavior, smoking, hypertension, hypercholesterolemia and diabetes. When stratified by age, sex and alcohol consumption, statistically significant positive associations were observed exclusively in participants aged ≥50 years, females and alcohol consumers. However, no significant interactions were detected across any subgroups (all P for interaction >0.05).

Figure 2B demonstrated significant positive associations between gallstones and osteoarthritis across multiple subgroups stratified by age, sedentary behavior, smoking status, hypertension, and hypercholesterolemia. When stratified by sex, BMI, alcohol consumption and diabetes, statistically significant positive associations were observed exclusively in females, those with BMI ≥ 25 kg/m^2^, alcohol consumers, and those without diabetes. Notably, interaction analysis revealed that sex significantly modified the association between gallstones and osteoarthritis(P for interaction = 0.02), indicating a particularly strong correlation in females.

Fig. 2C shows no significant associations between gallstones and rheumatoid arthritis in any subgroups, nor were any interaction effects observed (all P for interaction >0.05).

## 4 Discussion

This large-scale population-based study demonstrated a significant association between gallstones and arthritis, with particularly a robust effect observed for osteoarthritis. The persistence of this association after comprehensive adjustment for potential confounders suggested that gallstones are independently associated with arthritis, particularly osteoarthritis.

To our knowledge, this is the first study to investigate the association between gallstones and osteoarthritis. Although the pathophysiological relationship remains unclear, existing evidence provides several plausible mechanistic explanations centered on a shared metabolic-inflammatory axis. First, gallstone patients frequently exhibit systemic low-grade inflammation, even when asymptomatic(Psaltis et al., 2024). This may result from bacterial endotoxins (e.g., lipopolysaccharide) in the gallbladder mucosa triggering host inflammatory responses through Toll-like receptor 4/ nuclear factor kappa-light-chain-enhancer of activated B cells pathway activation, leading to elevated pro-inflammatory cytokines such as tumor necrosis factor-alpha, interleukin-1 beta and interleukin-18(Wang et al., 2023; Yu et al., 2024). Moreover, gallstone patients frequently present with metabolic disturbances including obesity, hyperlipidemia, and insulin resistance(Uche-Anya et al., 2024). These comorbidities may promote systemic inflammation via: (1) adipose tissue inflammation characterized by macrophage infiltration and elevated pro-inflammatory adipokines (e.g., leptin, resistin)(Codoñer-Franch and Alonso-Iglesias, 2015; Zhang et al., 2025); (2) cholesterol crystal-induced activation of the NOD-, LRR- and pyrin domain-containing protein 3 inflammasome pathway, leading to interleukin-1 beta overproduction(Duewell et al., 2010); (3) hyperglycemia/hyperinsulinemia-mediated oxidative stress through increased advanced glycation end products and mitochondrial dysfunction(Bellemare et al., 2025). This chronic low-grade inflammatory state, as evidenced in our study by elevated high-sensitivity C-reactive protein levels in gallstone patients (5.29 ± 8.64 vs 3.63 ± 6.98 mg/L, *P* < 0.01), may create a permissive environment for joint tissue degeneration through sustained activation of matrix metalloproteinases and inhibition of cartilage anabolic factors(Mukherjee and Das, 2024). Thus, systemic inflammation represents a plausible pathway linking gallstones to osteoarthritis. However, it is important to note that our cross-sectional design cannot determine whether systemic inflammation plays a causal mediating role in the association between gallstones and osteoarthritis, as the temporal sequence among variables remains unestablished.

Our subgroup and interaction analyses revealed a significant effect modification by sex (P for interaction = 0.02) in the association between gallstones and osteoarthritis. The association was substantially stronger in females (OR: 1.81, 95 % CI: 1.33, 2.47) than in males (OR: 1.02, 95 % CI: 0.66, 1.59). We hypothesize that this sexual dimorphism could be explained by hormonal influences. Estrogen has multifaceted effects on bile acid metabolism, including downregulation of bile acid transporters, modulation of bile acid synthetic enzymes, and promotion of cholesterol supersaturation in bile(Ismail et al., 2023; J. Wang et al., 2025a; Zu et al., 2021). The resulting bile acid metabolic disturbances may lead to impaired intestinal absorption of fat-soluble vitamin D(Thompson et al., 2023; Zhuang et al., 2024), causing deficiency in vitamin D's anti-inflammatory effects, cartilage protection and bone homeostasis(Fenercioglu, 2024; Guan et al., 2024; Montemor et al., 2025). This pathway represents a plausible biological mechanism that might contribute to the observed association in females.

On the other hand, our findings diverge from previous studies as we did not observe a significant association between gallstones and rheumatoid arthritis after adjusting for potential confounders (OR: 1.45, 95 % CI: 0.89, 2.36)(García-Gómez et al., 2019; Pamuk et al., 2006). This discrepancy may stem from several methodological distinctions: First, our study utilized a large, nationally representative community-based sample from NHANES, which provides greater statistical power and generalizability compared to previous studies predominantly conducted in specialized rheumatology clinic populations. Second, the community-based design minimizes selection bias inherent in hospital-based studies. Third, differences in the selection of adjusted confounding factors and variations in the study populations may also contribute to these discrepancies. Given these findings, the relationship between gallstones and rheumatoid arthritis requires cautious interpretation.

Our findings have potential clinical implications, although their causal relationship remains uncertain. The robust association between gallstones and osteoarthritis suggests that individuals diagnosed with gallstones, particularly in females, could be considered a potential target population for enhanced awareness, screening, and early intervention strategies for osteoarthritis. Regarding rheumatoid arthritis, the non-significant association after adjustment suggests no direct clinical implications, though it indirectly underscores the value of comprehensive metabolic management in rheumatic diseases.

Despite our findings, this study has several limitations. First, owing to the cross-sectional design, we could only establish an association between gallstones and arthritis rather than infer causality. The possibility of reverse causation cannot be excluded. For instance, systemic inflammation in rheumatoid arthritis or reduced physical activity due to painful osteoarthritis could potentially influence the risk of gallstones formation. Second, although our analysis adjusted for multiple covariates, residual confounding by unmeasured factors might still influence the observed correlation. Third, both gallstone and arthritis diagnoses were self-reported via questionnaires, potentially introducing selection bias. Fourth, this study was confined to the general US population, limiting generalizability to other ethnic groups. Thus, these limitations highlight the need for future prospective studies, including larger multi-ethnic cohorts, objective diagnostic measures and longitudinal assessment of temporal relationships of gallstone and arthritis to establish causality and clarify pathophysiological mechanisms.

## 5 Conclusions

This large-scale cross-sectional study based on NHANES data revealed a significant positive association between gallstones and arthritis, particularly osteoarthritis, with a more pronounced effect observed in females. The association remained robust even after adjusting for confounders, indicating an independent association between gallstones and osteoarthritis. In contrast, the association between gallstones and rheumatoid arthritis was not statistically significant after full adjustment. These findings indicated that individuals with gallstones, particularly in females, might benefit from heightened clinical attention for osteoarthritis.

## Prior presentation

This work has not been published before; it is not under consideration for publication anywhere else. This work been approved by all co-authors.

## CRediT authorship contribution statement

**Junyu Huang:** Writing – original draft, Visualization, Validation, Software, Methodology, Investigation, Formal analysis, Data curation. **Zan Liu:** Writing – review & editing, Supervision, Methodology.

## Consent for publication

Not applicable.

## Ethics approval and consent to participate

This study was conducted in accordance with the Declaration of Helsinki. The protocol was reviewed and approved by National Center for Health Statistics. Written informed consent was obtained from all participants prior to their inclusion in the study.

## Funding

No funding was received for conducting this study.

## Declaration of competing interest

The authors declare no competing interests.

## Data Availability

Data are publicly available online (https://wwwn.cdc.gov/nchs/nhanes/Default.aspx). The datasets used and analyzed during the current study are available from the corresponding author on reasonable request.
